# Progression of Low-Grade Neuroendocrine Tumors (NET) to High-Grade Neoplasms Harboring the NEC-Like Co-alteration of *RB1* and *TP53*

**DOI:** 10.1007/s12022-024-09835-y

**Published:** 2024-11-18

**Authors:** Nancy M. Joseph, Sarah E. Umetsu, Grace E. Kim, Merryl Terry, Arie Perry, Emily Bergsland, Sanjay Kakar

**Affiliations:** 1 Department of Pathology, University of California San Francisco (UCSF), 505 Parnassus Avenue, Room M-559, San Francisco, CA 94143 USA; 2 Department of Medicine, Division of Hematology/Oncology, University of California, San Francisco, CA USA

**Keywords:** High-grade neuroendocrine neoplasm, Neuroendocrine tumor, NET, Neuroendocrine carcinoma, NEC, p53, Rb, Genetics, Progression

## Abstract

High-grade or grade 3 epithelial neuroendocrine neoplasms (G3 NEN) are now divided into grade 3 well-differentiated neuroendocrine tumor (G3 NET) and neuroendocrine carcinoma (NEC), both defined by Ki-67 > 20% and/or > 20 mitoses per 2 mm^2^. NET and NEC are thought to be distinct tumors with different genetic profiles: NEC classically harbors co-alteration of *TP53* and *RB1*, whereas NET genetics are site-dependent with frequent alterations in *MEN1*, *ATRX*, *DAXX*, and *TSC1/2* in pancreatic NETs. Progression from NET to NEC is considered rare and is not well described. While both *TP53* and *RB1* alterations were initially thought to be rare in NET, recent work has demonstrated the former in up to 35% of high-grade G3 NET and the latter in rare high-grade NEN that progressed from NET. Here, we describe the clinical, pathologic, and molecular features associated with tumor evolution in a series of five patients that had low-grade NET that progressed to high-grade NEN with co-alteration of *RB1* and *TP53*, similar to NEC. Morphology of the high-grade neoplasms remained well-differentiated in some cases despite *RB1*/*TP53* co-alteration and had some NEC-like features in other cases. All five patients died of disease, with a mean overall survival of 41 months from the first metastatic disease and 12 months from acquisition of *RB1/TP53* co-alteration. Our data demonstrate that low-grade NET can progress via the acquisition of both *TP53* and *RB1* alteration, similar to NEC, but whether this represents a transformation from NET to NEC remains unclear.

**Supplementary Information:**

The online version contains supplementary material available at 10.1007/s12022-024-09835-y.

## Introduction

There are two main groups of epithelial neuroendocrine neoplasms (NENs): well-differentiated neuroendocrine tumor (NET) and poorly differentiated neuroendocrine carcinoma (NEC) [1, 2]. Based on the 2017 WHO, NET and NEC are distinct biological entities, thought to arise from different precursors and demonstrate distinct clinical, pathologic, and molecular features [3–12]. Progression from NET to NEC is considered rare and has not been clearly demonstrated. Rather, G3 NET are thought to progress from and share genetics with G1/G2 NET. NEC, on the other hand, are thought to arise de novo or in association with adenocarcinoma or squamous cell carcinoma of different organ sites. NECs of all organ sites have a uniformly poor prognosis and treatment almost always includes platinum-based chemotherapy and etoposide with or without radiation, surgery, and/or immunotherapy [13]. G3 NETs, on the other hand, are generally more indolent than NECs, although this group remains poorly understood, in part due to the evolving terminology and challenges in diagnosis, thus presenting challenges when performing retrospective studies using population-based or institutional registries.

Recent papers have demonstrated morphologic overlap between G3 NET and NEC, and the distinction between G3 NET and NEC, particularly large cell NEC, is a known diagnostic challenge [4, 14–17]. The morphologic features of G3 NET and NEC were not clearly teased out when the 2017 WHO divided NEC into these two groups. Both G3 NET and NEC still share the same grading cutoffs of a Ki-67 proliferation index of > 20% and/or a mitotic count > 20 per 2 mm^2^, and although G3 NET typically show lower Ki-67 indices and fewer mitoses than NEC, there is no formal upper limit of Ki-67 index or mitotic count for G3 NET. While G3 NEN that exhibit typical well-differentiated morphology (organoid growth, hyalinized stroma, uniform round to ovoid nuclei with only mild nuclear atypia) can be easily classified as G3 NET and those with classic small cell features (high nuclear-to-cytoplasmic ratio, enlarged hyperchromatic nuclei with molding, frequent apoptosis, brisk mitotic rate, scant cytoplasm) are readily classified as NEC, many G3 NEN cases show morphologic overlap between G3 NET and large cell NEC (growth in larger or irregular nests, increased nuclear atypia and pleomorphism, foci of necrosis, and desmoplasia). One study endeavored to establish a detailed set of morphologic criteria to separate G3 NET from NEC, with only 1.5% of cases being morphologically ambiguous; however, when these cases were later sequenced, there was discordance in the genetic profiles with the G3 NET group showing *RB1* alterations in 30% of cases [18, 19]. Other studies acknowledge ambiguous morphology in 34–60% of G3 NEN [4, 14, 16] and recommend integrating additional information to classify these cases. Several authors, including the 2019 and 2022 WHO panels, recommend that diagnostically ambiguous G3 NEN with prior history or co-existence of a low-grade NET should automatically be classified as G3 NET, essentially excluding the possibility of progression from NET to NEC [1, 5, 17]. Similarly, those with a prior history or co-existence of adenocarcinoma or squamous cell carcinoma would favor a diagnosis of NEC. For ambiguous cases without prior or co-existing precursor neoplasia, additional genetic testing and/or immunohistochemistry (IHC) is recommended.

The WHO 2017 states that the separation of NETs and NECs into sharp families is supported by genomic data. NECs demonstrate frequent co-alteration of *TP53* and *RB1* irrespective of site and additionally often show alterations common to adenocarcinoma or squamous cell carcinoma of the particular site (such as in *KRAS* and *SMAD4* for the pancreas) [12, 20–22]. The alterations in NET vary by site with pancreatic NETs demonstrating frequent alterations in *MEN1*, *DAXX*, *ATRX*, *TSC1*, *TSC2*, *CDKN1A*, and *CDKN1B*, as well as recurrent alterations in *CDKN2A* and *SETD2* in metastasis; *RB1* and *TP53* alterations were absent or rare in these studies [6, 8, 10–12, 17, 23–25]. Thus, based on these genomic data, IHC for p53 and Rb has been recommended in the distinction of G3 NET from NEC and has been shown to reduce interobserver variability in diagnosis [4, 14–17]. However, more recent molecular studies of G3 pancreatic NET have demonstrated higher rates of *TP53* alterations (10–38%), indicating that *TP53* can be acquired in NET progression and p53 IHC may not reliably distinguish NEC from G3 NET [14, 25–27]. Furthermore, rare G3 NEN that progressed from G1/G2 NET have been shown to harbor co-alteration of *TP53* and *RB1* in addition to alterations in *MEN1* and *ATRX*, suggesting progression from NET to NEC [14, 27]. Whether these cases should be classified as NEC because of aberrant p53 and Rb or G3 NET because they progressed from low-grade NET has not been addressed, and there is no definite data about treatment response or outcome for such cases.

The current study characterizes the clinical, morphologic, and molecular features of multiple tumor specimens from five patients who initially had low-grade G1/G2 well-differentiated NET that underwent grade progression accompanied by the acquisition of prototypical NEC co-alteration of *RB1* and *TP53*.

## Materials and Methods

### Case Selection

The UCSF pathology in-house and consult databases were searched for all cases of high-grade NEN (G3 NET, NEC, or high-grade NEN not otherwise specified) that had either a concurrent or history of prior low-grade NET. We identified five patients that had prior or concurrent low-grade NET as well as high-grade NENs demonstrating loss of Rb and aberrant p53, either by IHC or next-generation sequencing. All available pathology material for all five patients was reviewed including all available H&E and IHC slides from the primary NET resections for patients 1–4 (patient 5 had an unresected unknown primary). The pancreas primaries for patients 1–3 were sampled at one section per centimeter (cm) of the tumor, and the pituitary primary for patient 4 was entirely submitted for microscopic evaluation. All available pathology material for metastatic disease was also reviewed for all five patients (shown in Table 1). Data from the liver metastasis from patients 2 and 3 were reported in our prior study [14]; however, the prior study did not include any data from the primaries for these patients or the lymph node metastasis from patient 3. Furthermore, the prior study did not include any detailed clinical history or description of tumor evolution for these patients. Patient 4 is also reported as patient 6 in the paired paper on aggressive pituitary NENs by Terry et al. [28], but the molecular IHC illustrated in this manuscript for patient 4 was not illustrated in the paired paper.

**Table 1:**
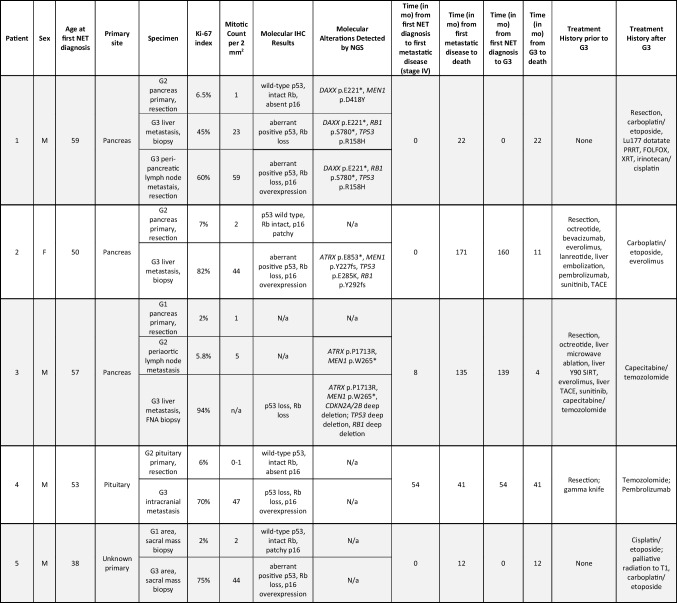
Clinical, pathologic, and molecular features of NET progression in five patients

-N/a is not available

### Immunohistochemistry

IHC for synaptophysin (polyclonal, Cell Marque), chromogranin A (LK2H10, Cell Marque), p53 (clone DO-7, Leica Biosystems), Rb (clone G3-245, BD Biosciences), p16 (clone E6H4, Roche mtm Laboratories), pan-cytokeratin (AE1/AE3, DAKO, and CAM5.2 BD Biosciences), CK7 (clone OV-TL12/30, DAKO), CAM5.2 (clone CAM5.2, BD Biosciences), TPIT (clone CL6251, Sigma), ATRX (polyclonal, Sigma), and Ki-67 (clone MIB1, DAKO) was performed on a representative whole-slide section of tumor from each case. If not already performed during the clinical work-up, missing IHC stains were performed on both low-grade NET and high-grade NEN from each patient. Ki-67 index was determined by manual count of 500 tumor cells in both low-grade and high-grade areas. For keratin, synaptophysin, chromogranin, and INSM1 staining, expression was scored as follows: (a) diffuse positive: staining in greater than 50% of tumor cells; (b) patchy positive: staining in 20–49% of cells; and (c) focal positive: staining in < 20% of cells. For p53, the staining pattern was interpreted as follows: (a) wild-type (patchy weak to moderate nuclear staining in < 50% of tumor cell nuclei), (b) loss (complete absence of staining in tumor cell nuclei and cytoplasm with appropriate staining internal control), (c) aberrant positive (≥ 60% of tumor cell nuclei strongly positive), or (d) equivocal (patchy moderate to strong staining in < 60% of nuclei—more than typical wild-type but less than clear cut positive). For p16, staining was designated as (a) overexpression (diffuse strong cytoplasmic or nuclear and cytoplasmic staining, also called “block-like” staining in essentially all tumor cells) [29], (b) patchy (variable positive p16 staining in a subset of cells), or (c) absent (complete absence or near complete absence of p16 staining with at most a cytoplasmic blush or rare positive cells); absent was not interpreted as lost/aberrant because many normal tissues show limited p16 staining.

### DNA Capture-Based Next-Generation Sequencing (NGS)

NGS was performed on a subset of the specimens in the context of routine clinical care, using the UCSF500 Cancer Gene panel, as previously described [14, 20, 30, 31].

## Results

Clinical, pathologic, and molecular features of low-grade NET and high-grade metastases from five patients are summarized in Table 1 and Supplemental Table 1. Tumors from all patients demonstrated diffuse keratin and synaptophysin expression, as well as focal, patchy, or diffuse expression of another neuroendocrine marker (chromogranin and/or INSM1). The primary NET site was the pancreas in three patients (patients 1–3), the pituitary in one patient (patient 4), and unknown in one patient (patient 5). The median patient age at initial NET diagnosis was 53 (range 38–59 years old). Four patients were male and one was female. Patients 1 and 5 had both low-grade NET and G3 NEN present at the time of initial diagnosis, without receiving any NET-related therapy prior to high-grade progression. Patients 2–4, on the other hand, received multiple NET-related treatments prior to high-grade progression, which occurred many years after the initial low-grade NET diagnosis. All five patients developed metastatic disease and died of disease. Three patients (patients 1, 2, and 5) had liver metastases at the time of the first NET diagnosis; patient 3 developed liver metastasis 8 months after the first diagnosis; patient 4, with a pituitary primary, developed intracranial metastasis 54 months after the first NET diagnosis. Median overall survival from the time of the first metastatic disease to the time of death was 41 months (range 12–171). The median overall survival from the time of G3 NEN diagnosis with Rb loss and aberrant p53 to the time of death was 12 months (range 4–41). Detailed descriptions of tumor progression are described for each patient below.

### Pancreatic Cases (Patients 1–3)

**Fig. 1:**
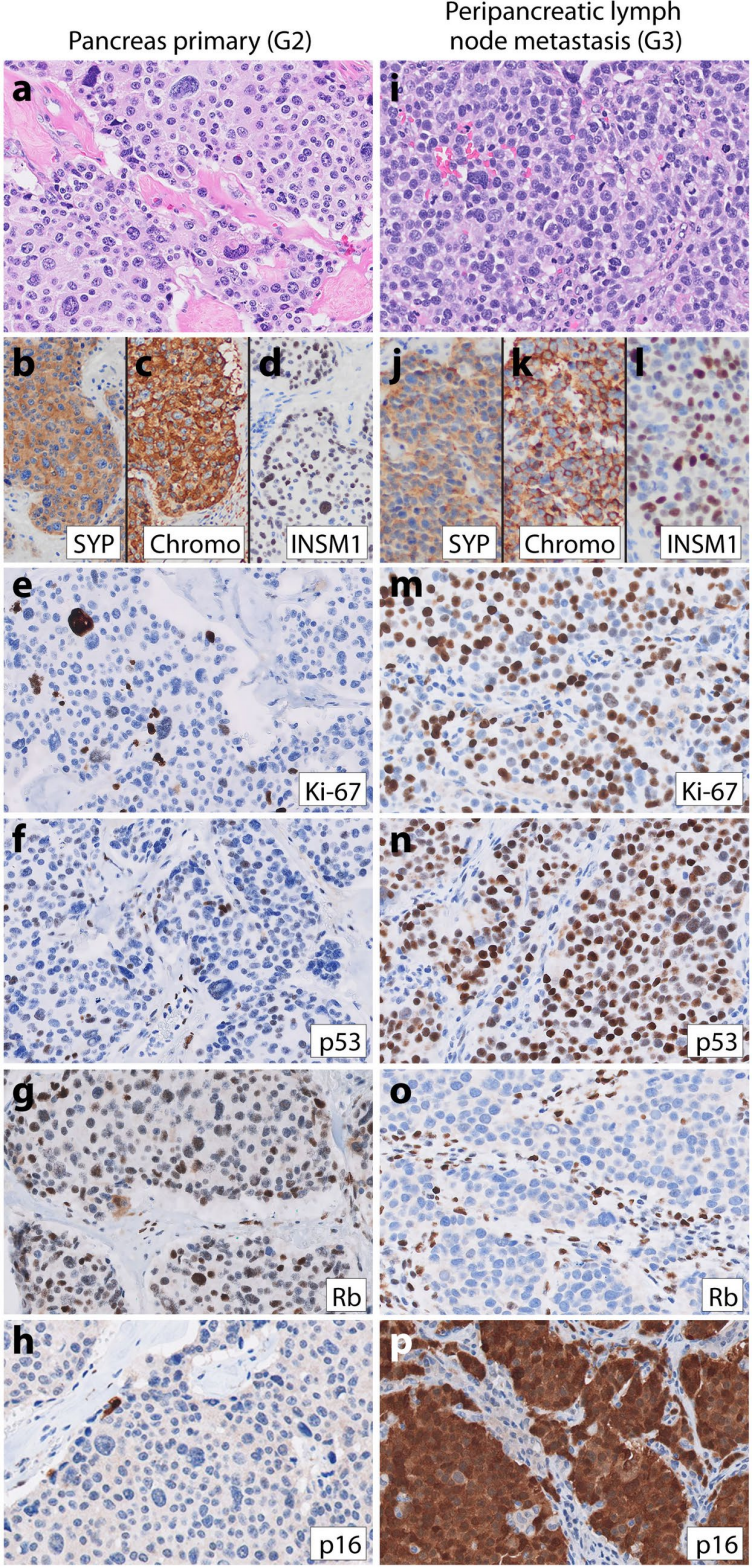
Representative images from patient 1’s low-grade primary pancreatic NET (**a**–**h**) and high-grade peri-pancreatic lymph node metastases (**i**–**p**). **a** High-magnification H&E image of the patient’s well-differentiated G2 NET in the pancreas shows the tumor growing in irregular variably sized nests with hyalinized stroma. Tumor cells had predominantly round to ovoid nuclei with salt and pepper chromatin, inconspicuous nucleoli, and rare mitoses, but there was notable pleomorphism with some tumor cells exhibiting larger, more atypical, irregular, and vesicular nuclei, known as degenerative atypia or endocrine atypia. IHC on the pancreas G2 NET demonstrated diffuse expression of **b** synaptophysin, **c** chromogranin, and **d** INSM1. **e** A Ki-67 index of 6.5%. The G2 NET also demonstrated **f** wild-type p53 staining, **g** intact Rb expression, and **h** essentially absent p16 expression. A high-grade peripancreatic lymph node metastasis (**i**–**p**) still maintained a predominantly nested growth pattern though some nests were larger and irregular as seen in panel **i** and tumor cellularity was increased with a higher nuclear-to-cytoplasmic ratio. Tumor cell nuclei were still predominantly round to ovoid but were slightly larger and darker than the G2 NET in the pancreas and mitoses were frequent; scattered large pleomorphic cells were still present. This high-grade lymph node metastasis also demonstrated diffuse expression for **j** synaptophysin, **k** chromogranin, and **l** INSM1 and had a **m** Ki-67 index of 60%, **n** aberrant-positive p53 staining, **o** loss of Rb expression, and **p** overexpression of p16

Patient 1, a 59-year-old male, presented with abdominal pain and weight loss. He was found to have a 15 cm heterogeneously enhancing pancreatic mass and multiple liver masses on a CT scan. Biopsy of a liver mass demonstrated a high-grade NEN with well-differentiated nested morphology, oncocytic cytoplasm, uniform nuclei, a mitotic count of 23 per 2 mm^2^, and Ki-67 index of 45%, suggestive of G3 NET, but IHC demonstrated aberrant p53 positivity and loss of Rb expression, suggestive of NEC (Supplemental Fig. 1). The patient was started on carboplatin and etoposide followed by Lu177-dotatate PRRT prior to pancreatectomy. Sections of the bulky tumor in the pancreas demonstrated variable morphologies: approximately half of the tumor was highly sclerosed, other areas were trabecular, and other areas demonstrated nested architecture within the hyalinized stroma and scattered pleomorphic nuclei, but mitoses were rare (Fig. 1; Supplemental Fig. 1). In the pleomorphic areas, the Ki-67 index was 6.5%, p53 staining was wild-type, Rb protein was intact, and p16 staining was absent (Fig. 1e–h), consistent with G2 NET. High-grade metastases were identified in two peri-pancreatic lymph nodes. The metastases had nested architecture, but some nests were larger and irregular; tumor cellularity, nuclear-to-cytoplasmic ratio, and mitoses were markedly increased compared with the primary pancreas tumor, and nuclear atypia was also increased (higher nuclear size, more hyperchromasia) (Fig. 1i). Thus, this neoplasm had some NEC-like features, but also some NET-like features (nested architecture), and so was considered morphologically ambiguous. One lymph node metastasis had a Ki-67 index of 60% and mitotic count of 59 per 2 mm^2^ and, like the liver metastasis, demonstrated aberrant positive p53, loss of Rb, and p16 overexpression (Fig. 1m–p). Sequencing of the G2 NET pancreas primarily demonstrated a nonsense mutation in *DAXX* (p.E221*) and a missense mutation in *MEN1* (p.D418Y). Sequencing of the G3 liver metastasis and the G3 lymph node metastasis both showed the identical DAXX mutation (p.E221*) as seen in the G2 pancreas NET. Both liver and lymph node metastases also harbored identical variants in *RB1* (p.S780*) and *TP53* (p.R158H) not seen in the G2 pancreas NET, which correlated with the Rb loss and aberrant positive p53 seen by IHC in both G3 specimens. Interestingly, the *MEN1* variant was not seen in either G3 neoplasm, suggesting loss of the mutant *MEN1* locus on chromosome 11q in the G3 neoplasms. None of the three samples was hypermutated. Postsurgical treatment included FOLFOX, radiation, and irinotecan plus cisplatin, but the patient died 8 months after surgery (22 months after initial diagnosis).

**Fig. 2:**
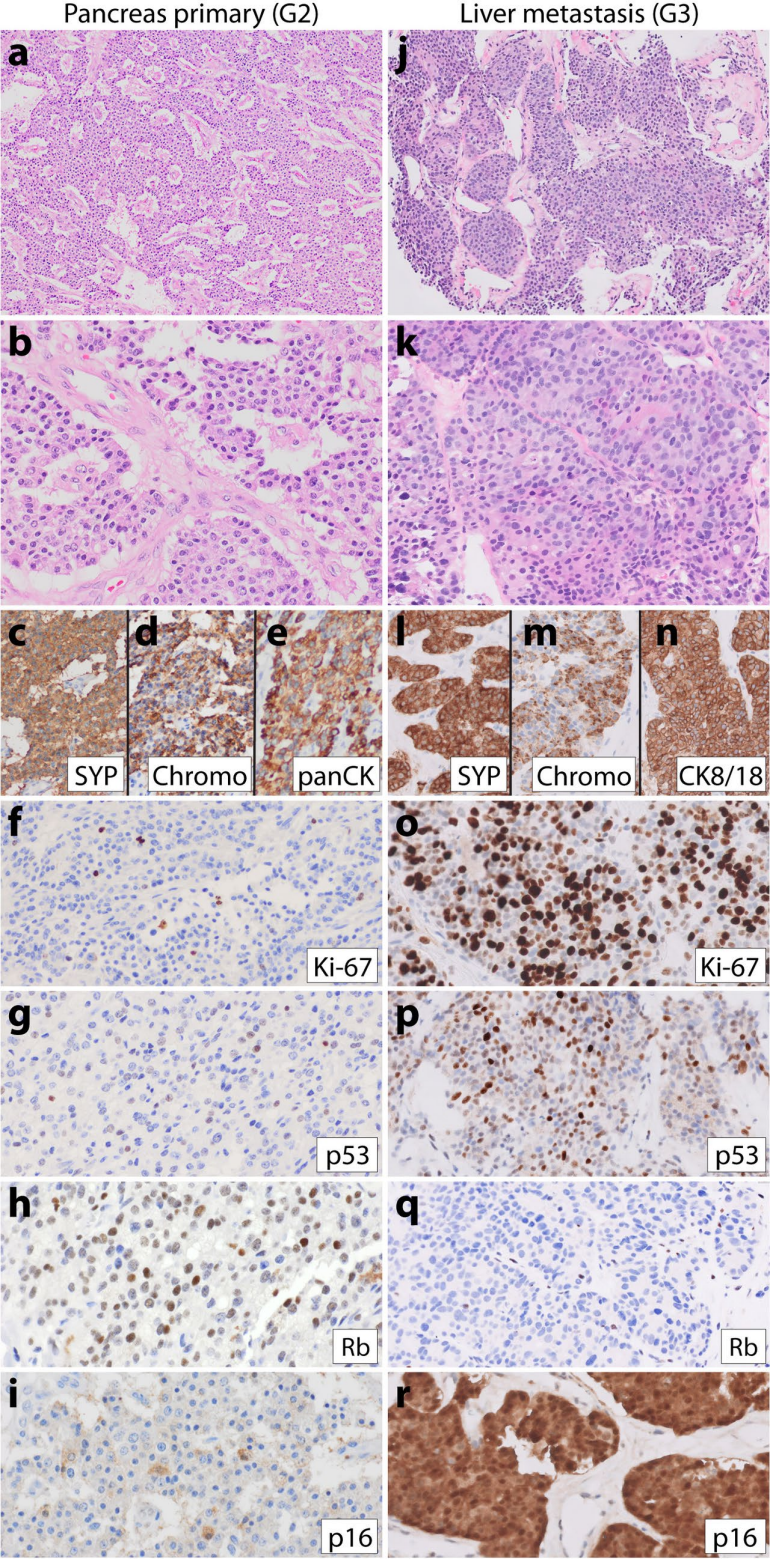
Representative images from patient 2’s low-grade primary pancreatic NET (**a**–**i**) and high-grade liver metastasis (**j**–**r**). Patient 2’s low-grade G2 pancreatic NET demonstrated trabecular architecture (**a**–**b**), diffuse **c** synaptophysin, **d** chromogranin, **e** keratin expression, and **f** Ki-67 proliferative index of 7%. IHC for **g** p53 was wild-type, **h** Rb was intact, and **i** p16 was patchy. A high-grade liver metastasis was biopsied 13 years later and demonstrated **j**–**k** similar well-differentiated morphology with trabecular and nested architecture and moderate eosinophilic cytoplasm, uniform ovoid nuclei with small nucleoli and frequent mitoses. IHC demonstrated diffuse **l** synaptophysin, **m** chromogranin, and **n** cytokeratin 8/18 expression. This liver metastasis had **o** Ki-67 index of 82%, **p** aberrant positive p53 staining, **q** loss of Rb expression, and **r** p16 overexpression

Patient 2, a 50-year-old female, had a longstanding history of pancreatic G2 NET with liver metastases since the time of initial NET diagnosis. High-grade progression was seen on the biopsy of a fast-growing liver metastasis 13.3 years after initial diagnosis. She received numerous treatments for NET in the years preceding high-grade progression, including surgical resection, octreotide, bevacizumab, everolimus, lanreotide, liver embolization, pembrolizumab, sunitinib, and transarterial chemoembolization (TACE). Sections of the high-grade neoplasm (Fig. 2j–k) showed well-differentiated morphology with nested and trabecular architecture, delicate vasculature, and monotonous ovoid nuclei resembling the initial primary pancreatic G2 NET (Fig. 2a–b), with the only significant difference on H&E stain being a brisk mitotic count of 44 mitoses per 2 mm^2^. Immunohistochemistry performed on the pancreas primary and high-grade liver metastasis demonstrated a marked increase in the Ki-67 labeling index from 7% in the primary to 82% in the G3 NEN. Furthermore, while the primary had wild-type p53, intact Rb, and patchy p16 (Fig. 2g–i), the high-grade liver metastasis demonstrated aberrant positive p53, loss of Rb, and overexpression of p16 (Fig. 2p–r). Sequencing of the G3 liver metastasis demonstrated mutations in *ATRX*, *MEN1*, *TP53*, and *RB1* without hypermutation. The patient was treated with carboplatinum and etoposide and then everolimus after high-grade progression, but passed away 11 months later.

**Fig. 3:**
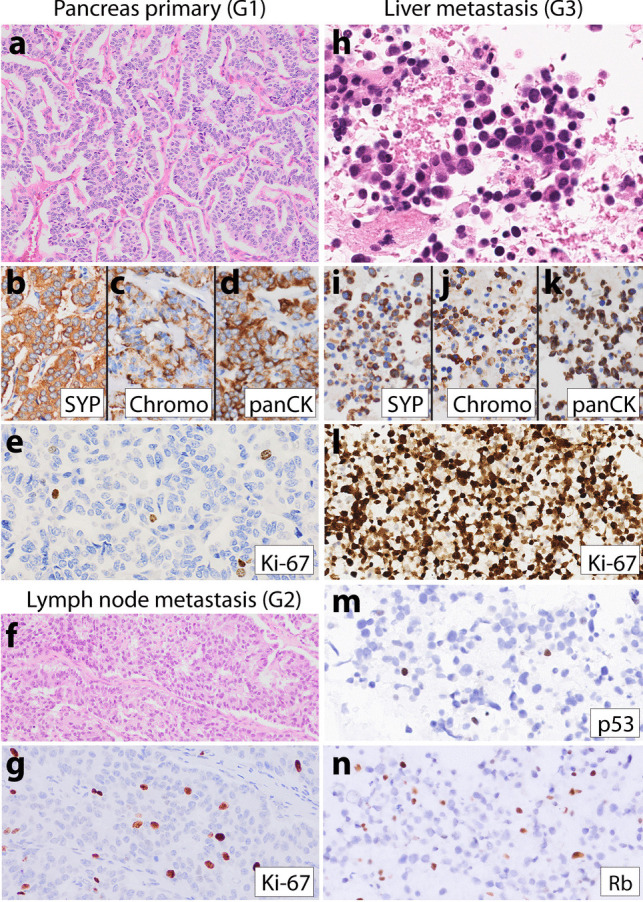
Representative images from patient 3’s low-grade primary pancreatic NET (**a**–**e**), low-grade lymph node metastasis (**f**–**g**), and high-grade liver metastasis (**h**–**n**). The low-grade G1 pancreatic NET had a **a** trabecular growth pattern, diffuse **b** synaptophysin, **c** chromogranin, **d** keratin expression, and **e** Ki-67 index of 2%. Eight months after the pancreas resection, the patient had a periaortic lymph node resection that demonstrated **f** well-differentiated NET with **g** Ki-67 index of 5.8%. Twelve years later, a fine needle aspiration biopsy of a liver metastasis demonstrated a high-grade neuroendocrine neoplasm with limited material that was difficult to interpret, though at least some of the tumor cells were plasmacytoid with moderate eosinophilic cytoplasm (**h**). IHC demonstrated diffuse-positive staining for **i** synaptophysin, **j** chromogranin, and **k** keratin. Although mitoses were not very frequent in the cell block, the Ki-67 index was 94% (**l**). Additional IHC demonstrated loss of both **m** p53 and **n** Rb expression in neoplastic cells

Patient 3, a 57-year-old man, similarly had a longstanding history of metastatic pancreatic low-grade NET many years prior to high-grade progression. His initial primary pancreatic NET was G1 at diagnosis (Fig. 3a–e); his first lymph node (Fig. 3f–g) and liver metastases were G2 when resected 8 months after his pancreas resection and 11 years prior to having high-grade progression documented on fine needle aspiration (FNA) biopsy of a fast-growing liver metastasis (Fig. 3h–n). He received numerous treatments for NET in the years preceding high-grade progression, including surgical resection, octreotide, liver microwave ablation, liver Y90 selective internal radiation therapy (SIRT), everolimus, liver TACE, sunitinib, and capecitabine/temozolomide. The FNA cell block was scant and difficult to evaluate as the neoplastic cells were extensively dissociated. Some neoplastic cells appeared to have plasmacytoid morphology typical of NET (Fig. 3h), but overall, we considered this case not informative regarding morphology due to the limited material. Immunohistochemistry on the cell block demonstrated a Ki-67 index of 94% and a loss of both p53 and Rb (Fig. 3l–n). Sequencing of the G3 liver metastasis demonstrated mutations in *ATRX* and *MEN1* and deep deletions in *CDKN2A/2B*, *TP53*, and *RB1*. Sequencing of a G2 periaortic lymph node metastasis from 11 years prior to high-grade progression demonstrated identical variants in *ATRX* and *MEN1*, but no alterations in *TP53* or *RB1*. Neither sample was hypermutated. The patient was treated with capecitabine and temozolomide after high-grade progression but died of the disease 4 months later.

### Pituitary Case (Patient 4)

**Fig. 4:**
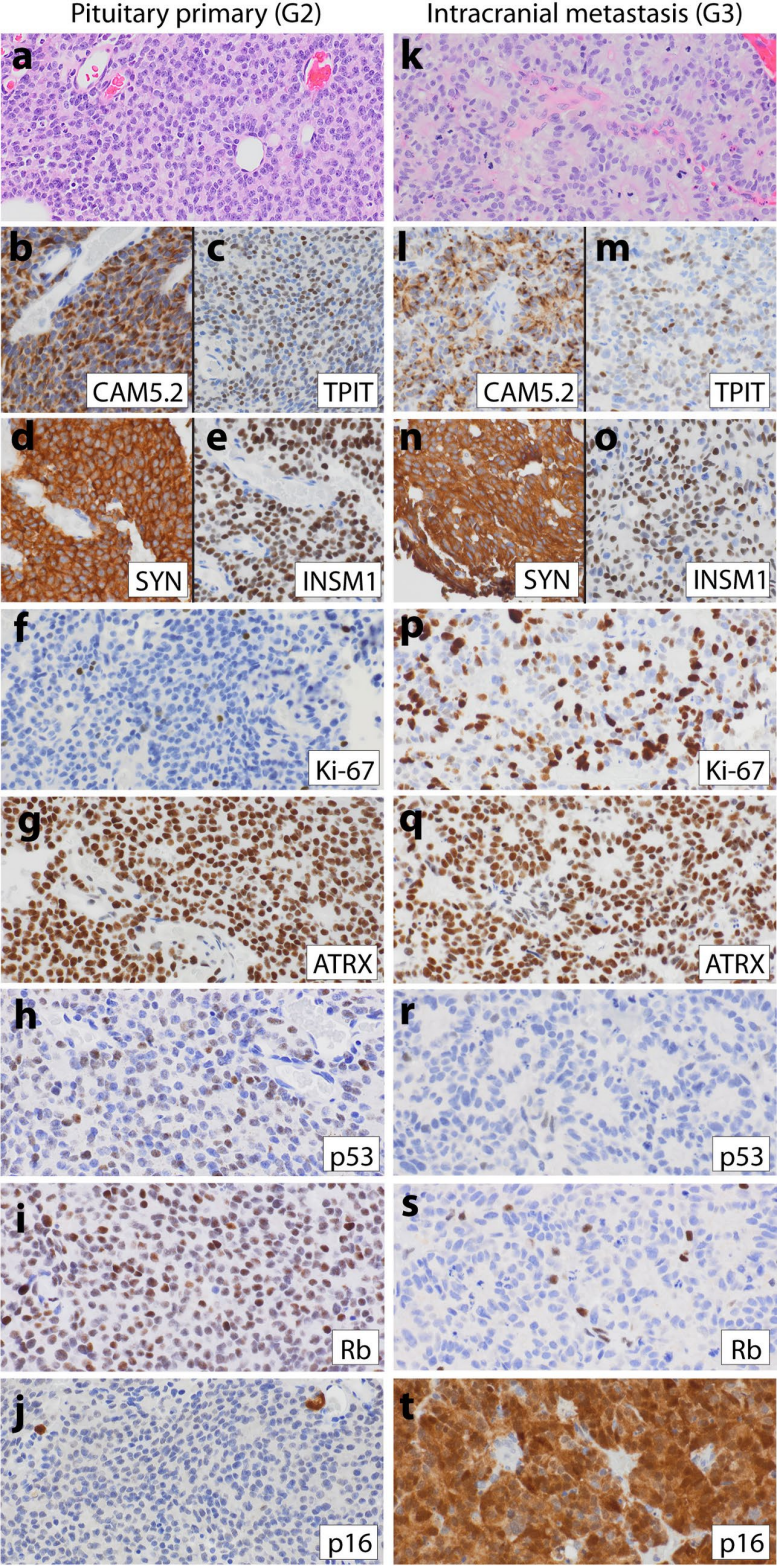
Representative images from patient 4’s low-grade primary pituitary NET (**a**–**j**) and high-grade intracranial metastasis (**k**–**t**).** a** High-magnification H&E image of patient 4’s initial well-differentiated pituitary NET demonstrates tumor growth in sheets with occasional rosettes, delicate vasculature, and uniform round to ovoid nuclei with inconspicuous nucleoli and rare mitoses. IHC demonstrated significant positivity for **b** keratin, **c** TPIT, **d** synaptophysin, **e** INSM1, and **f** Ki-67 index of 6%. The low-grade NET also demonstrated **g** intact ATRX expression, **h** wild-type p53 staining, **i** intact Rb expression, and **j** absent p16. Patient 4 had a resection of an **k**–**t** intracranial metastasis 4.5 years after initial diagnosis, which had **k** similar morphologic features to the original pituitary NET, but with more numerous rosettes, mitoses, and apoptotic nuclei. The metastasis demonstrated **l** diffuse keratin expression with a dot-like staining pattern and **m** positive but more limited TPIT expression than the primary. **n**–**o** Neuroendocrine markers expression was diffuse, and **p** Ki-67 index was 70%. **q** ATRX expression was intact; both **r** p53 and **s** Rb expression were lost, and **t** p16 was overexpressed

Patient 4, a 53-year-old man, underwent resection of a 2.2-cm pituitary mass. Diagnostic workup of the initial resection revealed a low-grade pituitary NET with ACTH expression. Prior to high-grade progression seen in an intracranial metastasis 4.5 years later, the patient was treated with additional resections, as well as Gamma knife radiosurgery. The initial pituitary NET (Fig. 4a–j) demonstrated sheets/trabeculae of uniform epithelioid cells with delicate vasculature, occasional rosettes, rare mitoses, Ki-67 index of 6%, wild-type p53, intact Rb, and absent p16 expression. The patient’s fourth resection (4.5 years later) demonstrated similar well-differentiated morphology though with more numerous rosettes, frequent mitoses, elevated Ki-67 index of 70%, loss of p53 and Rb, and p16 overexpression (Fig. 4k–t). After this high-grade progression, the patient survived for an additional 41 months and was treated with temozolomide and pembrolizumab, but eventually developed cerebrospinal fluid (CSF) metastases and passed away.

### Unknown Primary (Patient 5)

**Fig. 5:**
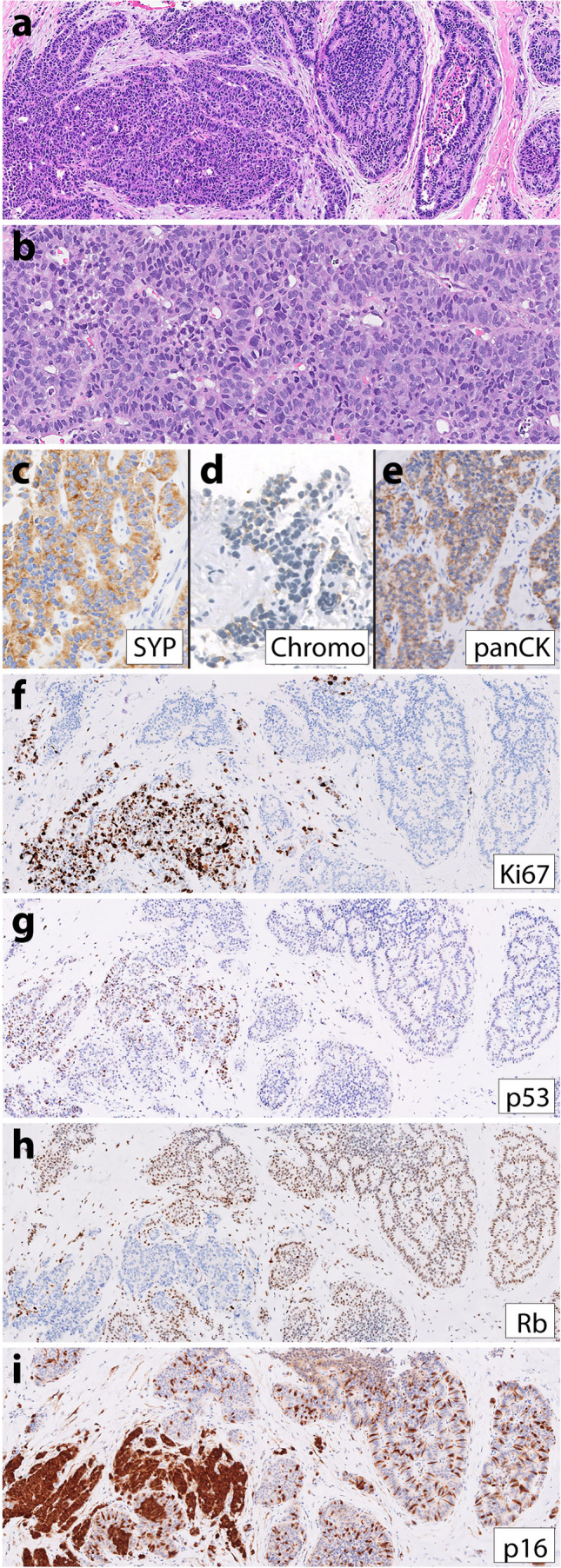
Representative images from patient 5’s presacral mass biopsy with both low-grade and high-grade areas. **a** and **f**–**i** H&E and IHC stains of patient 5’s presacral mass biopsy demonstrating a well-differentiated G1 NET with growth in nests on the right side and a high-grade NEN focus on the left side with a more trabecular but irregular and infiltrative growth pattern. **b** A high-magnfiication image of the high-grade component only demonstrating increased nuclear atypia characterized by enlarged nuclei with irregular borders, smudgy chromatin, and frequent mitoses. Both components had **c** diffuse synaptophysin, **d** focal chromogranin, and **e** diffuse keratin expression. The low-grade component had **f** Ki-67 index of 2%, **g** wild-type p53, **h** intact Rb, and **i** patchy-positive p16 expression (wild-type pattern). The high-grade focus on the left side demonstrated **c** a marked increase in the Ki-67 index at 75%, **d** aberrant-positive p53 expression, **e** loss of Rb expression, and **f** overexpression of p16

Patient 5, a 38-year-old male with Currarino syndrome (germline *MNX1* mutation), presented with low back pain and was found to have a pre-sacral mass as well as liver metastases. Biopsy of the pre-sacral mass demonstrated a predominantly G1 NET with a distinct focus of high-grade progression (Fig. 5). The G1 NET component had organoid morphology with growth in nests and ribbons, a Ki-67 proliferation index of 2%, intact Rb, wild-type p53, and patchy p16 staining. The higher-grade component showed increased nuclear atypia (Fig. 5b), proliferation index of 75%, loss of Rb, aberrant positive p53, and p16 overexpression. The high-grade focus had some NEC-like morphologic features; it was surrounded by desmoplastic stroma, and tumor cells had a higher nuclear-to-cytoplasmic ratio and larger more atypical nuclei with increased mitoses compared with the low-grade areas. However, the high-grade tumor cells still had some NET-like architectural features with growth in trabeculae/ribbons, thus still vaguely organoid. Overall, the G3 focus was considered morphologically ambiguous. Similar to patient 1, patient 5 demonstrated G3 NEN progression at the time of initial diagnosis and therefore did not receive any NET-related treatments prior to progression. This patient was treated with cisplatin and etoposide, palliative radiation, and carboplatin/etoposide after diagnosis, but died 12 months post-diagnosis.

## Discussion

High-grade NEN consists of G3 NET and NEC, which have different biology, clinical behavior, and therapies. However, these entities can exhibit overlapping morphologic and/or genetic features making final classification and optimal treatment uncertain in some cases. Progression from NET to NEC is thought to be rare and is not well described. Our study shows that low-grade NETs can undergo high-grade progression involving the acquisition of alterations in both *TP53* and *RB1*, the prototypical co-alteration seen in NEC. In all cases in this series, the low-grade NET lacked p53 and Rb abnormalities, while all corresponding high-grade NEN exhibited aberrant p53 and Rb based on IHC and/or sequencing. In addition to *TP53/RB1* alterations, mutations typical of NETs like in *DAXX, MEN1*, and *ATRX* were also present in two progressed high-grade neoplasms; these were identical variants as seen in the corresponding low-grade NETs, supporting a common origin of both the low-grade and high-grade tumors. While previous studies have demonstrated that 0–10% of pancreatic NET (G1–G3 combined) and around one-third of G3 pancreatic NET can show mutations in *TP53* without *RB1* alterations, co-alteration of *TP53* and *RB1* is rare. Our previous study reported *TP53* and *RB1* co-alteration in two G3 pancreatic NEN with a history of prior low-grade NET though the corresponding low-grade NETs were not evaluated in this study to prove clonality [14]. The recent study by Kasajima et al. in this issue reports a single G3 NEN with a history of prior rectal NET with co-alteration of *TP53* and *RB1* [27].

The morphology of the low-grade NETs in all five patients was typical for well-differentiated NET, with either nested or trabecular architecture (Supplemental Table 1). Patient 1 is the only patient in our series with morphologic variants that have recently been suggested to have prognostic value. The primary pancreas NET had pleomorphic foci (Fig. 1), which are reportedly less aggressive, and the high-grade lymph node metastasis displayed pleomorphism as well. The high-grade liver metastasis had oncocytic features, which are reportedly more aggressive [32]. This case demonstrates that clinically aggressive cases can show variable morphologies, including one reported to be less aggressive.

Despite high-grade progression with co-acquisition of *TP53* and *RB1* aberrations, three of the high-grade NEN (patient 1 liver metastasis, patient 2, and patient 4) maintained well-differentiated NET morphology, but with striking increases in both Ki-67 proliferative indices and mitotic counts. These results indicate that aberrant p53 and Rb, considered the hallmark of NEC, can be seen in tumors with morphologic features of NET. Although not included in the official definitions of NET and NEC in the WHO (2017, 2019, and 2022), the use of IHC and/or genetics has been recommended for diagnosis [4, 14, 16]. It is not clear if high-grade tumors with NET morphology (G3 NETs by WHO) with Rb or Rb/p53 alterations behave as G3 NET or NEC.

Morphology was ambiguous in two high-grade neoplasms that progressed from NET (patient 1 lymph node metastasis and patient 5). It remains unclear if high-grade NENs with ambiguous or large cell NEC-like morphologic features that progressed from low-grade NETs and show both NET-related mutations as well as *RB1/TP53* mutations deserve classification as G3 NET or NEC. It is possible that NEC is biologically heterogeneous with a small subset arising from the progression of NETs, while the majority arise de novo or in association with adenocarcinoma or another type of non-neuroendocrine carcinoma. It has been suggested that cases that morphologically resemble NEC but have progressed from NET should be classified as G3 NET irrespective of morphology, but at that time, NET progression with *TP53* and *RB1* co-alteration had not been described. We recommend designating these tumors as high-grade NEN until additional data becomes available. Progression of low-grade NET to morphologically unequivocal small cell NEC was not identified in our study and has not yet been reported in the literature to our knowledge.

Whether this series of cases represents the transformation of NET to NEC and whether these should be treated like NEC remains to be determined. All five patients died after high-grade progression; three died within a year of high-grade progression similar to NEC outcomes; one survived 22 months; and one patient, treated with temozolomide and pembrolizumab, survived 41 months after progression which is more similar to G3 NET outcomes [33]. Though based on a small number of cases, there was no clear correlation of morphologic features with outcome; one patient with well-differentiated morphology survived less than 1 year after high-grade progression, and the patient with the shortest overall survival of 4 months had non-informative morphology due to limited tissue. Furthermore, given the small size of this case series and the variable treatments employed, it is not possible to draw any meaningful conclusions about treatment options from this study. Larger studies are needed to determine the optimal therapy for these patients. Currently, the NET versus NEC treatment options for such borderline cases are dictated by a combination of other features such as response to prior therapy, extent of disease, rate of progression based on serial radiologic studies, Ki-67 proliferation index, and mutational profile.

In conclusion, the cases in these series raise important questions about the diagnosis and classification of high-grade NENs with overlapping morphology and genetics. NET can progress to a high-grade neoplasm with some NEC-like genetic (co-alteration in *TP53* and *RB1*) and morphologic features. Rb and p53 co-aberrations are typical of NEC but can rarely occur in neoplasms with NET morphology. Larger studies with appropriate control groups (G3 NET without *TP53/RB1* co-alterations and large cell NEC) are needed to determine the clinical outcomes, response to treatment, and best classification in these cases. We recommend designating these tumors as high-grade NEN or G3 NEN until additional data becomes available (see Table 2).

**Table 2:**
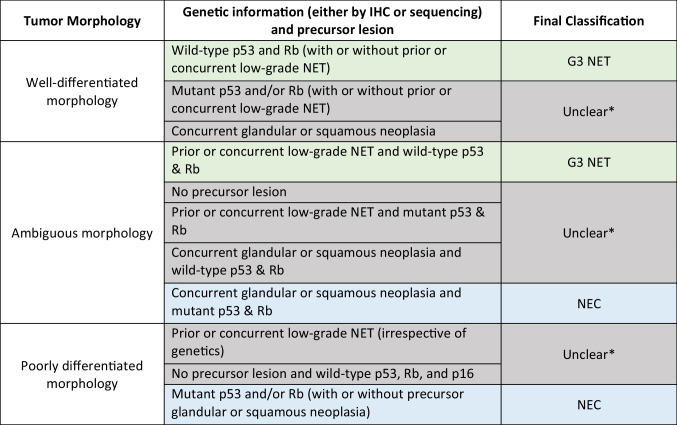
Spectrum of high-grade (grade 3) NEN including straightforward G3 NET, straightforward NEC, and cases with overlapping features between G3 NET and NEC

^*^Final classification remains unclear in cases that show some features of G3 NET but other features of NEC, as the natural history of these tumors is not fully understood. For such cases, we recommend a diagnosis of high-grade neuroendocrine neoplasm (G3 NEN) with a comment explaining the differential (G3 NET versus NEC) and the unclear final classification due to overlapping features between G3 NET and NEC

## Supplementary Information

Below is the link to the electronic supplementary material.

Supplementary file1 (DOCX 14 KB)

Supplemental Figure 1. Additional images from patient 1 include heterogeneous morphologies of the low-grade pancreas primary and images of the high-grade liver metastasis. Patient 1 had a large 15-cm pancreatic primary that was sampled at one section per cm. Sections demonstrated **a** pleomorphic areas also shown in Fig. 1, **b** sclerosing areas which represented approximately 50% of the tumor, and **c** more typical trabecular areas. **d** Pankeratin expression was diffuse in the pancreas NET. Interestingly, patient 1 had two high-grade NET metastases, which showed different morphologies. **e** H&E image from a high-grade liver metastasis with well-differentiated morphology, nested growth, and oncocytic features. Like the lymph node metastasis shown in Fig. 1, and here in panel **k** for comparison, the liver metastasis (**e**–**j**) had diffuse **f** synaptophysin and **g** chromogranin expression, **h** high Ki-67 index of 45%, as well as **i** aberrant positive p53 expression and **j** loss of Rb expression. However, the **e** high-grade liver metastasis demonstrated oncocytic morphology, not seen in the **k** high-grade lymph node metastasis. (PNG 4.04 MB)

High Resolution Image (TIFF 17.9 MB)

## Declarations

### Data Availability

Data from the liver metastasis from patients 2 and 3 were reported in our prior study [14]; however, the prior study did not include any data from the primaries for these patients or the lymph node metastasis from patient 3. Furthermore, the prior study did not include any detailed clinical history or description of tumor evolution for these patients. Patient 4 is also reported as patient 6 in the paired paper on aggressive pituitary NENs by Terry et al. [28], but the molecular IHC illustrated in Fig. 4 was not illustrated in the paired paper.
