# Supplementary material for: Progression of Low-Grade Neuroendocrine Tumors (NET) to High-Grade Neoplasms Harboring the NEC-Like Co-alteration of RB1 and TP53
Source: Endocr Pathol. 2024 Nov 18;35(4):325–37. doi: 10.1007/s12022-024-09835-y (PMC11659342; doi:10.1007/s12022-024-09835-y)
Supplement: Supplementary file 1 — Supplementary file1 (DOCX 14 KB) [file 12022_2024_9835_MOESM1_ESM.docx]

Supplemental Table 1: Morphology, keratin, and neuroendocrine marker expression in low-grade NET and subsequent high-grade NEN

| **Patient** | **Specimen** | **Morphologic Variants** | **Keratin*** | **Synaptophysin** | **Chromogranin** | **INSM1** |
| --- | --- | --- | --- | --- | --- | --- |
| **1** | Pancreas Primary (15 cm, G2) | 50% sclerosing; 50% trabecular or solid/nested with pleomorphic foci | Diffuse | Diffuse | Diffuse | Diffuse |
|  | Peripancreatic Lymph Node Metastasis (G3) | Irregular and solid/nested with pleomorphism | N/a | Diffuse | Diffuse | Diffuse |
|  | Liver Metastasis (G3) | Solid/nested; Oncocytic features | CK7: Diffuse | Diffuse | Diffuse | N/a |
| **2** | Pancreas Primary (6 cm, G2) | Trabecular | Diffuse | Diffuse | Diffuse | Diffuse |
|  | Liver Metastasis G3 | Solid/nested | CK8/18: Diffuse | Diffuse | Diffuse | N/a |
| **3** | Pancreas Primary (3.4 cm, G1) | Trabecular | Diffuse | Diffuse | Patchy | Patchy |
|  | Periaortic Lymph Node Metastasis (G2) | Trabecular and solid/nested | N/a | Diffuse | Patchy | N/a |
|  | Liver Metastasis (G3) | Not informative due to insufficient tissue | Diffuse | Diffuse | Diffuse | N/a |
| **4** | Pituitary primary (G2) | Trabecular | CAM5.2: Diffuse | Diffuse | N/a | Diffuse |
|  | Pituitary/ethmoid recurrence (G3) | Trabecular | CAM5.2: Diffuse, dot-like | Diffuse | N/a | Diffuse |
| **5** | Sacral metastasis (G1 area) | Nested | Diffuse | Diffuse | Focal | N/a |
|  | Sacral metastasis (G3 area) | Irregular and trabecular | Diffuse | Diffuse | Focal | N/a |
| *Pan-cytokeratin cocktail containing AE1/AE3/CAM5.2 was used unless a different cytokeratin is specified | | | | | | |
| -N/a is not available | |  |  |  |  |  |
