# Supplementary figures and images for: Progression of Low-Grade Neuroendocrine Tumors (NET) to High-Grade Neoplasms Harboring the NEC-Like Co-alteration of RB1 and TP53
Source: Endocr Pathol. 2024 Nov 18;35(4):325–37. doi: 10.1007/s12022-024-09835-y (PMC11659342; doi:10.1007/s12022-024-09835-y)

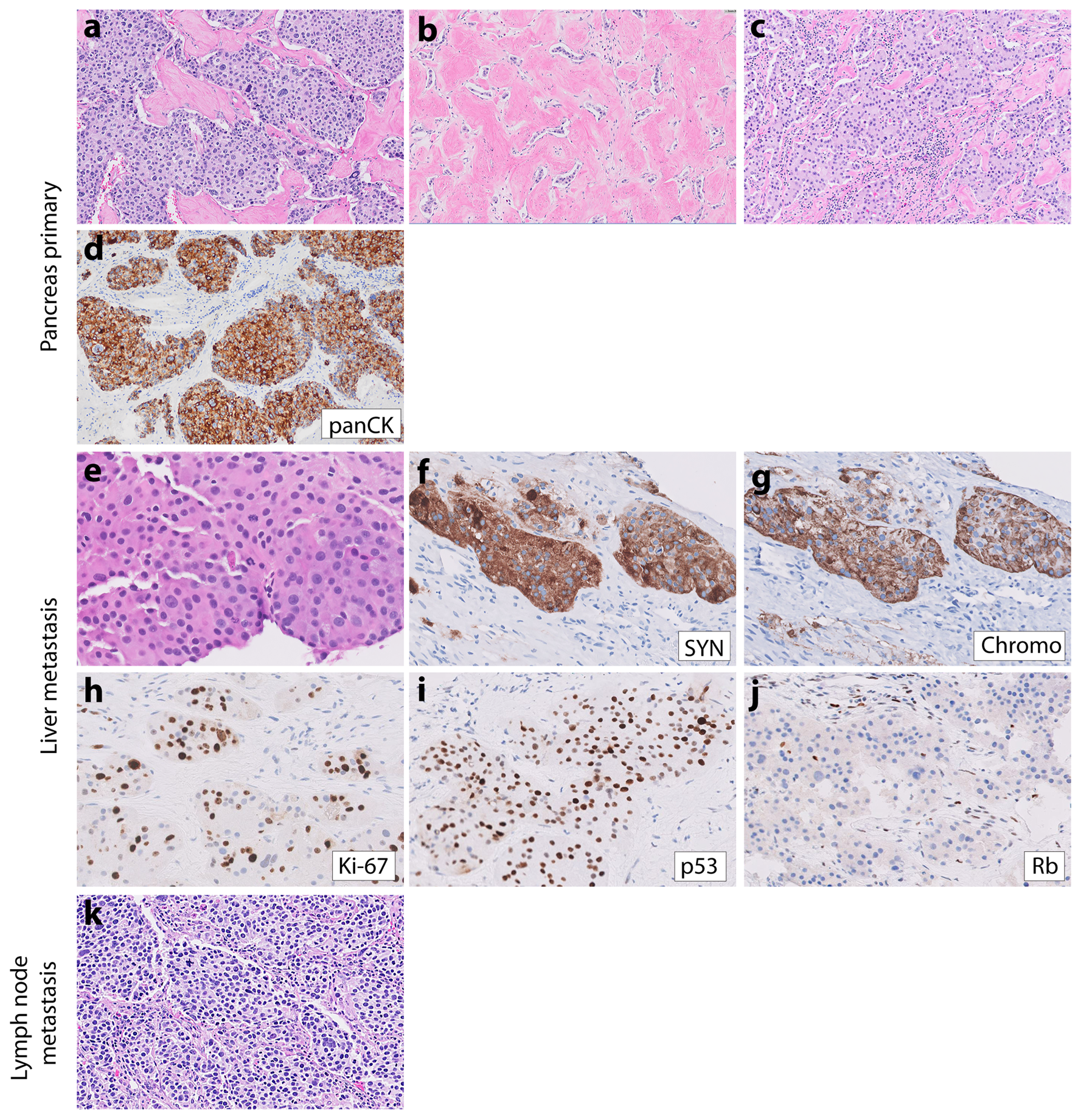

Supplement: Supplementary file 2 — Additional images from patient 1 include heterogeneous morphologies of the low-grade pancreas primary and images of the high-grade liver metastasis. Patient 1 had a large 15-cm pancreatic primary that was sampled at one section per cm. Sections demonstrated a pleomorphic areas also shown in Fig. 1, b sclerosing areas which represented approximately 50% of the tumor, and c more typical trabecular areas. d Pankeratin expression was diffuse in the pancreas NET. Interestingly, patient 1 had two high-grade NET metastases, which showed different morphologies. e H&E image from a high-grade liver metastasis with well-differentiated morphology, nested growth, and oncocytic features. Like the lymph node metastasis shown in Fig. 1, and here in panel k for comparison, the liver metastasis (e–j) had diffuse f synaptophysin and g chromogranin expression, h high Ki-67 index of 45%, as well as i aberrant positive p53 expression and j loss of Rb expression. However, the e high-grade liver metastasis demonstrated oncocytic morphology, not seen in the k high-grade lymph node metastasis. (PNG 4.04 MB) [file 12022_2024_9835_Fig6_ESM.png]

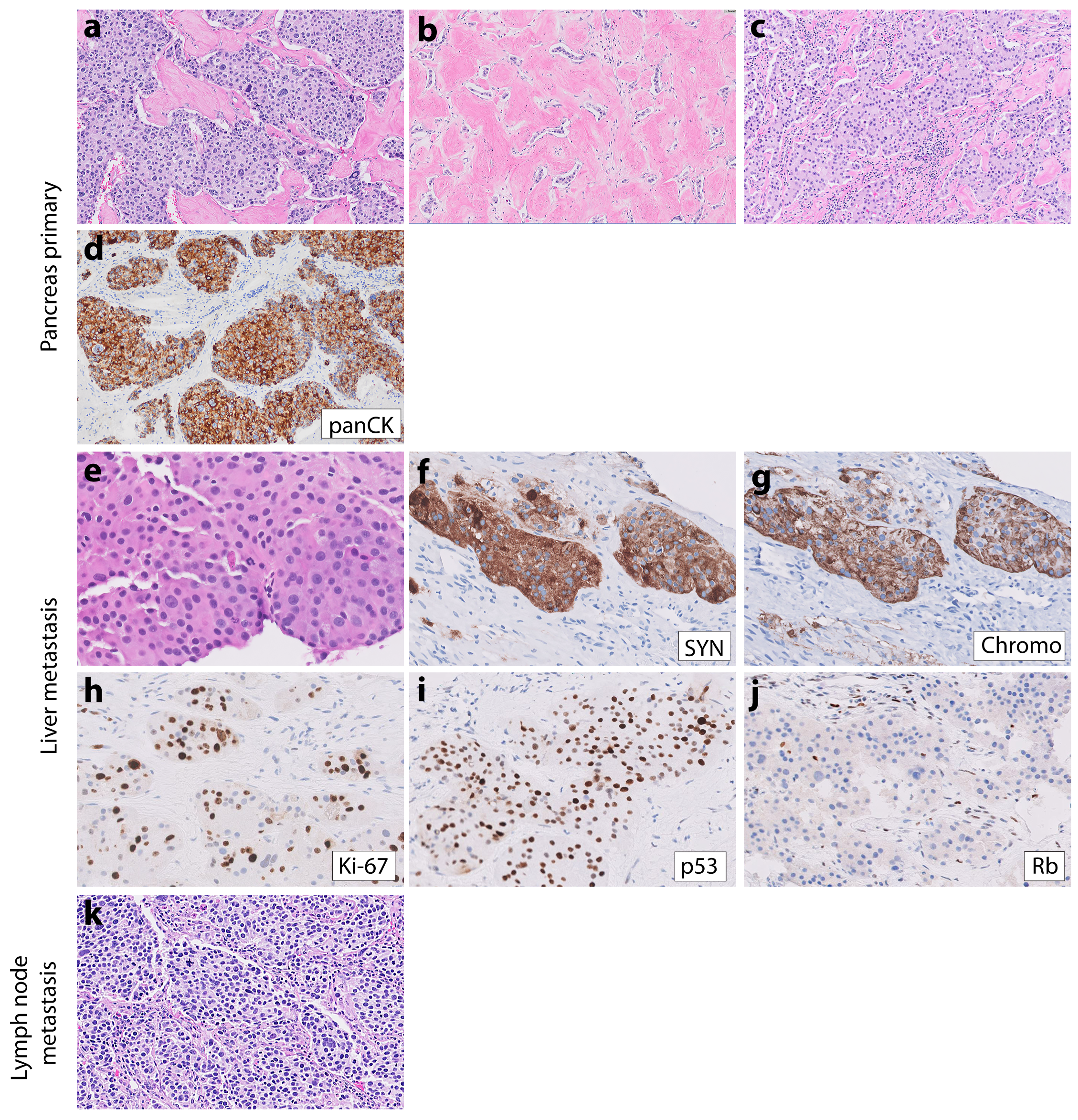

Supplement: Supplementary file 3 — High Resolution Image (TIFF 17.9 MB) [file 12022_2024_9835_MOESM2_ESM.tif]
